# Streptococcal pyrogenic exotoxin B inhibits apoptotic cell clearance by macrophages through protein S cleavage

**DOI:** 10.1038/srep26026

**Published:** 2016-05-16

**Authors:** Chia-Ling Chen, Yueh-Ying Wu, Chiou-Feng Lin, Chih-Feng Kuo, Chia-Li Han, Shuying Wang, Woei-Jer Chuang, Chiu-Yueh Chen, Jiunn-Jong Wu, Pei-Jane Tsai, Ching-Chuan Liu, Yee-Shin Lin

**Affiliations:** 1 Translational Research Center, Taipei Medical University, Taipei, Taiwan; 2 Department of Microbiology and Immunology, College of Medicine, National Cheng Kung University, Tainan, Taiwan; 3 Graduate Institute of Medical Sciences, College of Medicine, Taipei Medical University, Taipei, Taiwan; 4 Department of Microbiology and Immunology, College of Medicine, Taipei Medical University, Taipei, Taiwan; 5 Department of Nursing, I-Shou University, Kaohsiung, Taiwan; 6 Master Program for Clinical Pharmacogenomics and Pharmacoproteomics, Taipei Medical University, Taipei, Taiwan; 7 Center of Infectious Disease and Signaling Research, National Cheng Kung University, Tainan, Taiwan; 8 Department of Biochemistry and Molecular Biology, College of Medicine, National Cheng Kung University, Tainan, Taiwan; 9 Department of Medical Laboratory Science and Biotechnology, College of Medicine, National Cheng Kung University, Tainan, Taiwan; 10 Department of Pediatrics, National Cheng Kung University Hospital, Tainan, Taiwan

## Abstract

Clearance of apoptotic cells by macrophages plays an important role in maintaining tissue homeostasis. Previous study indicated that streptococcal pyrogenic exotoxin B (SPE B) reduces phagocytic activity in group A streptococcus (GAS) infection. Here, we demonstrate that SPE B causes an inhibitory effect on protein S-mediated phagocytosis. In the presence of SPE B, serum- and purified protein S-mediated phagocytosis of apoptotic cells were significantly inhibited. The binding abilities of protein S to apoptotic cells were decreased by treatment with SPE B. Bacterial culture supernatants from GAS NZ131 strain also caused a reduction of protein S binding to apoptotic cells, but *speB* mutant strain did not. SPE B directly cleaved protein S *in vitro* and *in vivo*, whereas a lower level of cleavage occurred in mice infected with a *speB* isogenic mutant strain. SPE B-mediated initial cleavage of protein S caused a disruption of phagocytosis, and also resulted in a loss of binding ability of protein S-associated C4b-binding protein to apoptotic cells. Taken together, these results suggest a novel pathogenic role of SPE B that initiates protein S degradation followed by the inhibition of apoptotic cell clearance by macrophages.

*Streptococcus pyogenes*, also called group A streptococcus (GAS), is an important human pathogen that causes various diseases with a wide range of severity, including pharyngitis, impetigo, scarlet fever, acute rheumatic fever, post-streptococcal glomerulonephritis, necrotizing fasciitis, and streptococcal toxic shock syndrome^1^. A number of bacterial virulence factors such as hyaluronic acid capsules, M proteins, C5a peptidase, streptolysins, and streptococcal pyrogenic exotoxins have been identified as playing roles in streptococcal diseases^2^. Among these, streptococcal pyrogenic exotoxin B (SPE B) is a critical virulence factor in the production of tissue damage and severe lethal effect in GAS-infected mouse models, and is also highly associated with toxic shock syndrome and mortality in patients^3^,^4^,^5^. However, SPE B has also been shown to be negatively regulated during invasive infections and the expression of active SPE B is inversely related to disease severity^6^,^7^,^8^.

SPE B, a cysteine proteinase, can degrade several host factors such as fibronectin, vitronectin, fibrinogen, matrix metalloprotease and E-cadherin to facilitate bacterial dissemination, colonization, and invasion^2^,^9^,^10^. In addition, SPE B also digests immunoglobulins, properdin as well as C3 to inhibit complement activation and neutrophil opsonophagocytosis, which help protect GAS against human defense mechanisms^9^,^11^,^12^,^13^,^14^. Mice infected with the *speB* isogenic mutant showed decreased resistance to phagocytic activity as compared with mice infected with the wild-type strain, which led to impaired bacterial dissemination, tissue damage, and increased mouse survival rate^15^,^16^. In addition to help bacteria to resist phagocytosis, SPE B can directly trigger apoptosis in various cells including macrophages and epithelial cells^17^,^18^. The involvement of SPE B in the clearance of apoptotic cells is less clear.

Apoptotic cell clearance by phagocytes (a process called efferocytosis) is an important mechanism to sustain cellular homeostasis. The deficiency in clearance may cause secondary necrosis of apoptotic cells and then elicit inflammatory responses and autoimmunity^19^,^20^,^21^. A number of factors and receptors expressed on the surface of phagocytes or present in serum are involved in mechanisms of apoptotic cell clearance^22^,^23^,^24^. Phosphatidylserine (PS) exposed early on the apoptotic cell surface, constitutes “eat me” signals for efferocytosis through PS receptor (PSR) recognition by phagocytes^19^,^23^,^24^. In addition to PS/PSR, previous studies also reported the requirement of serum-derived vitamin K-dependent protein S for the enhancement of apoptotic cell clearance by macrophages^25^. Protein S may act as a bridging molecule, which can bind PS and in turn be recognized by its relative receptor Tyro 3/Mer on macrophages to facilitate apoptotic cell engulfment^24^,^25^,^26^. Inflammatory response and autoantibodies are dramatically generated in aging mice with protein S deficiency^27^. Protein S not only functions as a cofactor of protein C in the anti-coagulation pathway, but also acts as an opsonin involved in bridging apoptotic cells with phagocytes^22^,^23^,^24^,^28^. Since macrophage phagocytosis represents an essential defense mechanism against GAS infection^29^, it is intriguing to ask whether GAS may modulate phagocytosis for apoptotic cell clearance and lead to disease progression.

SPE B has been reported to cause a decrease of opsonophagocytosis-mediated killing through immunoglobulin degradation and complement system disruption^11^,^12^,^13^,^14^. Moreover, SPE B directly triggers macrophages to undergo apoptosis thereby reducing macrophage phagocytosis^17^. These studies suggest an anti-phagocytic role of SPE B in GAS infection. Additionally, GAS infection is associated with substantial apoptosis^15^,^17^,^30^,^31^,^32^. Nevertheless, there is no report showing the regulation of apoptotic cell clearance during GAS infection. In the present study, we demonstrate a novel pathogenic action of SPE B on macrophage-mediated clearance of apoptotic cells through protein S degradation. Furthermore, protein S-mediated binding of C4b-binding protein (C4BP) to apoptotic cells is inhibited in the presence of SPE B. Theses results indicate that SPE B-regulated protein S degradation leads to a decrease of efferocytosis and also loses the protection of C4BP on apoptotic cells from complement attack, which may trigger secondary necrosis and severe inflammatory responses.

## Results

### Serum-mediated enhancement of apoptotic cell clearance by macrophages is suppressed by active SPE B

**Figure 1:**
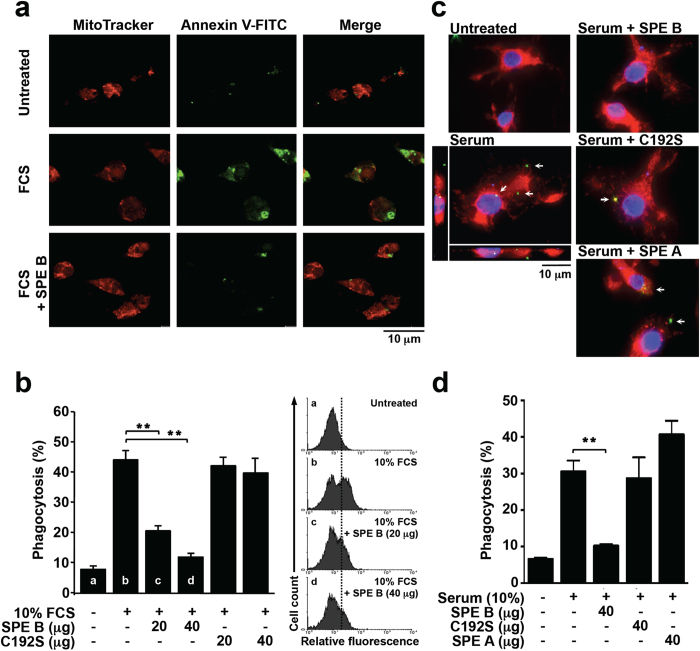
SPE B reduces the serum-mediated apoptotic cell phagocytosis by macrophages. (**a**) Freshly isolated CD11b-positive mouse peritoneal macrophages were cultured on sterile slide and stained with mitochondrial MitoTracker (*Red*). STS (100 μM)-stimulated apoptotic Jurkat T cells were prepared and stained with FITC-conjugated annexin V (*Green*). These two types of cells were then co-incubated with 10% FCS alone or FCS pretreated with 20 μg SPE B for 1 h at 37 °C. The engulfment of apoptotic cells by macrophages was determined using confocal microscopy. Scale bar is shown. (**b**) In the presence or absence of FCS and FCS pretreated with SPE B or protease-negative mutated protein C192S for 1 h, the percentages of apoptotic cell engulfment by macrophages were measured by flow cytometry (*left*). The histograms, (**a–d**), are shown (*right*). (**c**) PMA (160 nM)-stimulated THP-1 cells were stained with MitoTracker (*Red*) and incubated with FITC-labeled apoptotic cells (*Green*) in the presence or absence of 10% human serum and serum pretreated with SPE B, C192S, or SPE A followed by deconvolution microscopic analysis. Arrows indicate the ingested apoptotic cells. The Z-stacks are shown to the left (YZ) and bottom (XZ) of the main image (XY). Nuclear staining with DAPI (*Blue*) and the scale bars are shown. (**d**) In the presence of human serum and serum pretreated with SPE B, C192S, or SPE A for 1 h, the percentages of apoptotic cell engulfment by PMA-stimulated THP-1 cells were measured by flow cytometry. Data are shown as mean ± SD of three independent experiments. **P < 0.01 as compared with FCS or serum treatment alone.

In GAS infection, SPE B can directly degrade opsonins, such as immunoglobulin and complement, to decrease opsonophagocytic killing of bacteria^11^,^12^,^14^. Opsonins in the serum are also involved in the bridging between apoptotic cells and phagocytes to efficiently promote apoptotic cell clearance^22^,^23^,^28^. Previous studies have indicated the anti-phagocytic effects of SPE B^11^,^12^,^13^,^14^,^17^. We therefore speculated that serum-mediated phagocytosis of apoptotic cells may be regulated by SPE B. To characterize the role of SPE B in serum-mediated apoptotic cell engulfment by macrophages, purified recombinant SPE B was incubated with fetal calf serum (FCS) followed by phagocytic analysis. Jurkat T cells were first stimulated with staurosporine (STS) for 12 h^33^, and the apoptotic cells were then stained with FITC-labeled annexin V (~80%, Supplementary Fig. S1a). Macrophages were derived from mouse peritonea and their purity was confirmed by an antibody specific for CD11b (Supplementary Fig. S1b). By confocal microscopic observation, the presence of FCS promoted considerable apoptotic cell engulfment by macrophages, whereas SPE B-pretreated FCS caused less phagocytosis (Fig. 1a). Flow cytometric analysis also showed that the percentages of FCS-mediated phagocytosis were significantly reduced when FCS was pretreated with SPE B but not C192S, a SPE B mutated protein lacking protease activity (Fig. 1b). In order to further verify the effect of SPE B on human macrophage phagocytosis, we used PMA-stimulated THP-1 cells (Supplementary Fig. S1c). PMA-treated THP-1 cells ingested FITC-labeled apoptotic cells in the presence of human serum; however, when human serum was pretreated with SPE B, phagocytosis was inhibited (Fig. 1c). Furthermore, human serum pretreated with C192S or SPE A (identified as a streptococcal superantigen) still enhanced phagocytosis of apoptotic cells (Fig. 1c). In addition, the measurement of efferocytosis using flow cytometric analysis also revealed that SPE B but not C192S or SPE A impeded serum-mediated apoptotic cell phagocytosis by PMA-stimulated THP-1 cells (Fig. 1d). To avoid a direct apoptotic effect of SPE B on macrophages, we added broad protease inhibitor E64 to terminate the reactions of SPE B at indicated time points before co-incubation with macrophages and apoptotic cells. Previous studies indicated that serum complement augments the phagocytosis of apoptotic cells^34^. In our experiments, we used heat-inactivated serum (56 °C for 30 min) which still showed enhancement of phagocytosis. Therefore, SPE B may exert suppression of apoptotic cell phagocytosis by a complement-independent mechanism.

### SPE B disrupts protein S-mediated enhancement of apoptotic cell clearance by macrophages

**Figure 2:**
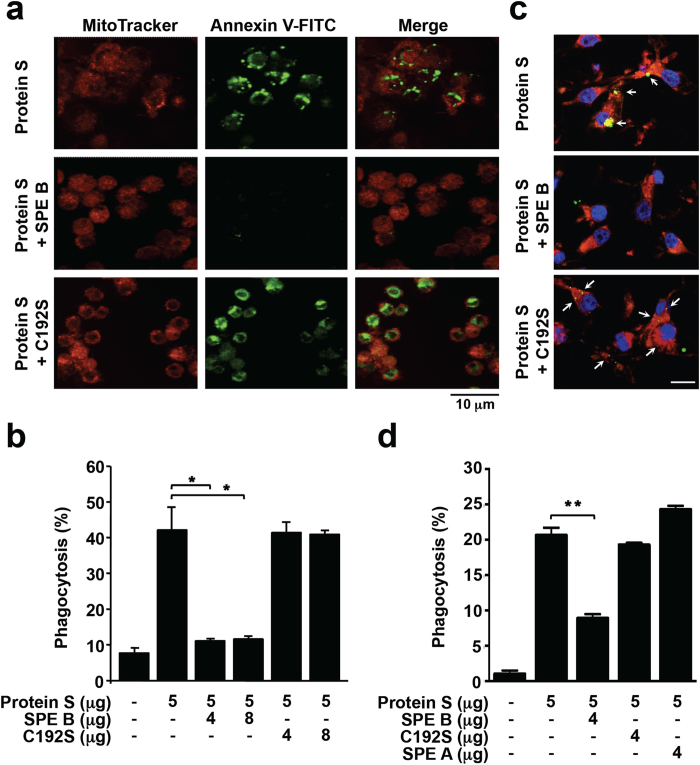
SPE B reduces protein S-mediated apoptotic cell phagocytosis by macrophages. (**a**) In the presence of 5 μg purified protein S, or protein S pretreated with 4 μg SPE B or C192S for 1 h, MitoTracker (*Red*)-stained mouse peritoneal macrophages were incubated with FITC-conjugated annxein V (*Green*)-labeled apoptotic Jurkat T cells for 1 h at 37 °C. The engulfment of apoptotic cells by macrophages was determined using confocal microscopy. Scale bar is shown. (**b**) In the presence or absence of protein S and protein S pretreated with different dosages of SPE B or C192S for 1 h, the percentages of apoptotic cell engulfment by macrophages were measured by flow cytometry. (**c**) The engulfment of apoptotic cells (*Green*) by PMA-stimulated THP-1 cells (*Red*) in the presence of protein S and protein S pretreated with SPE B or C192S were detected using confocal microscopy. Arrows indicate the ingested apoptotic cells. Nuclear staining with DAPI (*Blue*) and the scale bars are shown. (**d**) In the presence of protein S and protein S pretreated with SPE B, C192S, or SPE A for 1 h, the percentages of apoptotic cell engulfment by PMA-stimulated THP-1 cells were measured by flow cytometry. Data are shown as mean ± SD of three independent experiments. *P < 0.05 and **P < 0.01 as compared with protein S treatment.

Human plasma-derived vitamin K-dependent protein S has been demonstrated to be involved in regulating apoptotic cell clearance by macrophages^25^,^27^. It is intriguing to ask whether SPE B inhibits serum-stimulated phagocytosis by disrupting the action of protein S in the serum. To examine the effect of SPE B on protein S, purified recombinant SPE B or mutated protein C192S were incubated with purified protein S followed by phagocytosis analysis. By confocal microscopic observation, SPE B-pretreated protein S showed a decreased phagocytosis of apoptotic cells than protein S treatment alone (Fig. 2a). However, C192S-pretreated protein S still exhibited similar phagocytic activity as protein S treatment alone, suggesting that SPE B protease activity modulates protein S-regulated phagocytosis of apoptotic cells. For quantification, the percentages of Jurkat T cells engulfed by macrophages were measured by flow cytometry, and the results showed that SPE B but not C192S can directly and effectively disrupt protein S-mediated apoptotic cell clearance (Fig. 2b). Similarly, SPE B but not C192S (Fig. 2c) or SPE A (Supplementary Fig. S2) disrupted protein S-mediated apoptotic cell phagocytosis by PMA-stimulated THP-1 cells. Furthermore, SPE B-mediated inhibition in efferocytosis was also detected in PMA-stimulated THP-1 cells using flow cytometric analysis, whereas C192S or SPE A did not cause an effect (Fig. 2d).

### SPE B reduces the binding of protein S to apoptotic cells

**Figure 3:**
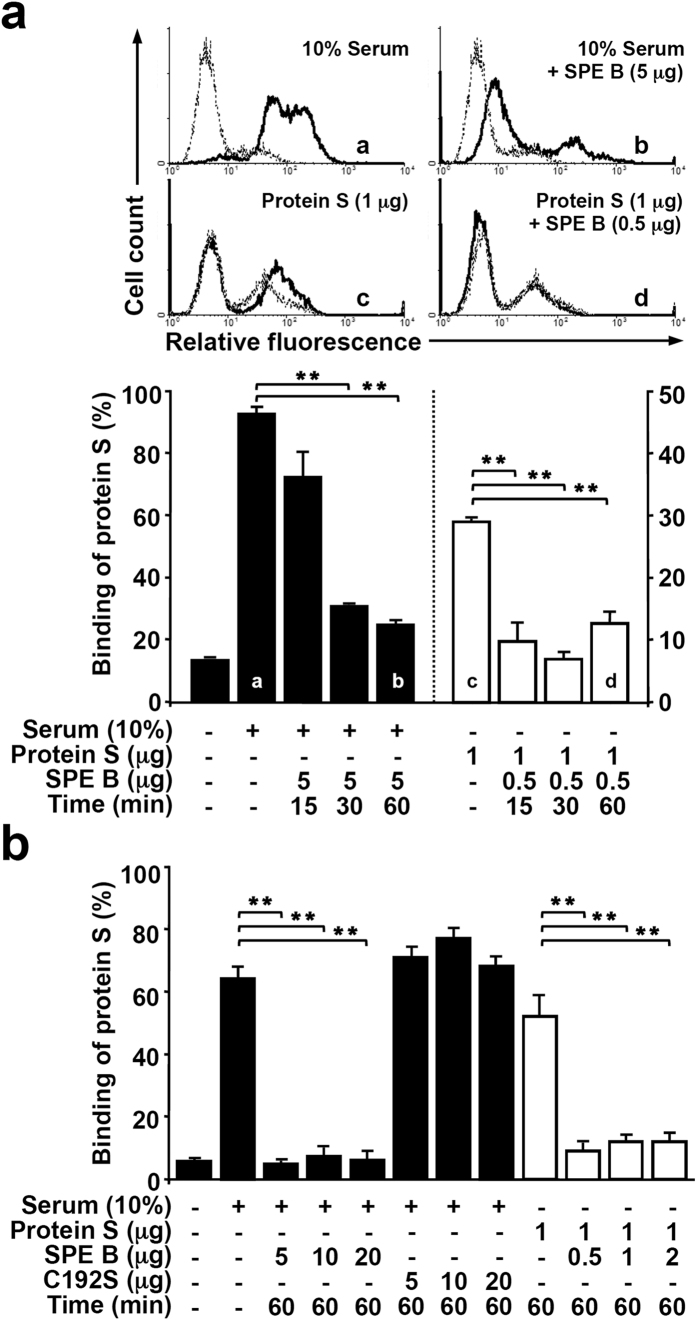
SPE B reduces the binding ability of protein S on apoptotic cells. (**a**) Human serum, protein S, or SPE B-pretreated human serum or protein S at the indicated time points were incubated with STS-stimulated apoptotic cells for 25 min followed by binding analysis. The percentages of protein S binding to apoptotic cells were measured using the nonspecific antibody (*dotted line*) or the specific antibody against protein S (*solid line*) staining followed by flow cytometric analysis. The histograms (*upper)*, a–d, and quantified results are shown (*lower*). Data are shown as mean ± SD of three independent experiments. **P < 0.01 as compared with serum or protein S treatment. (**b**) In the presence or absence of human serum, protein S, human serum pretreated with different dosages of SPE B or C192S, and protein S pretreated with different dosages of SPE B, the bindings of protein S to apoptotic cells were detected using flow cytometric analysis. Data are shown as mean ± SD of three independent experiments. **P < 0.01 as compared with serum or protein S treatment.

Protein S displays a high-affinity binding to PS expressed on the surface of apoptotic cells, and promotes a specific recognition through the relevant receptors on the phagocytes^22^,^23^,^24^,^25^,^28^. Since pretreatment of protein S with SPE B could markedly affect protein S-mediated phagocytosis, we further verified the binding ability of protein S to apoptotic cells in the presence of SPE B. Human serum or purified protein S were mixed with SPE B for different time points followed by incubation with apoptotic Jurkat T cells. The binding percentages of protein S on Jurkat T cells were subsequently measured by staining with specific antibody against human protein S. Results showed that both human serum and purified protein S could bind to apoptotic Jurkat T cells, whereas pretreatment with SPE B caused an inhibition of binding (Fig. 3a). In contrast, C192S did not show inhibition as that of SPE B (Fig. 3b). These results thus demonstrate that SPE B but not C192S directly disrupts protein S binding to apoptotic cells and the subsequent attenuation of phagocytosis.

### GAS-secreted SPE B suppresses protein S binding to apoptotic cells

**Figure 4:**
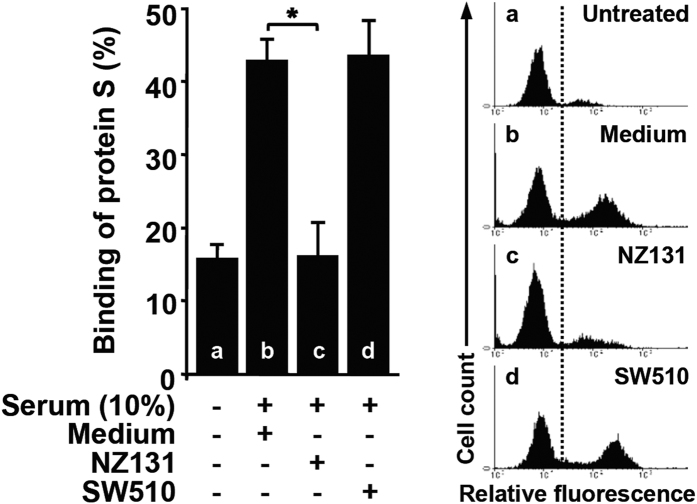
GAS-secreted SPE B inhibits protein S binding to apoptotic cells. Bacterial culture supernatants were collected from *speB* wild-type strain NZ131 and *speB* mutant strain SW510 that were incubated in RPMI growth medium at 37 °C for 18 h. To determine the binding activity of protein S to apoptotic cells, human serum was first incubated with medium or bacterial culture supernatants for 1 h, which were subsequently co-incubated with STS-stimulated apoptotic cells at room temperature for 25 min followed by the specific protein S staining and flow cytometric analysis. The histograms, a–d, and their percentages of protein S-binding cells are shown as mean ± SD of three independent experiments. **P < 0.05 as compared with the medium-treated group.

To further evaluate the effects of SPE B secreted from GAS on the function of protein S, bacterial culture supernatants collected from wild-type strain NZ131 and *speB* mutant strain SW510 (no SPE B) were used. Results indicated that bacterial culture supernatants from NZ131 significantly inhibited the binding of protein S derived from human serum with apoptotic Jurkat T cells, whereas SW510 did not (Fig. 4). Therefore, GAS-secreted SPE B also causes disruption of protein S binding to apoptotic cells.

### Active SPE B causes protein S degradation and dysfunction

**Figure 5:**
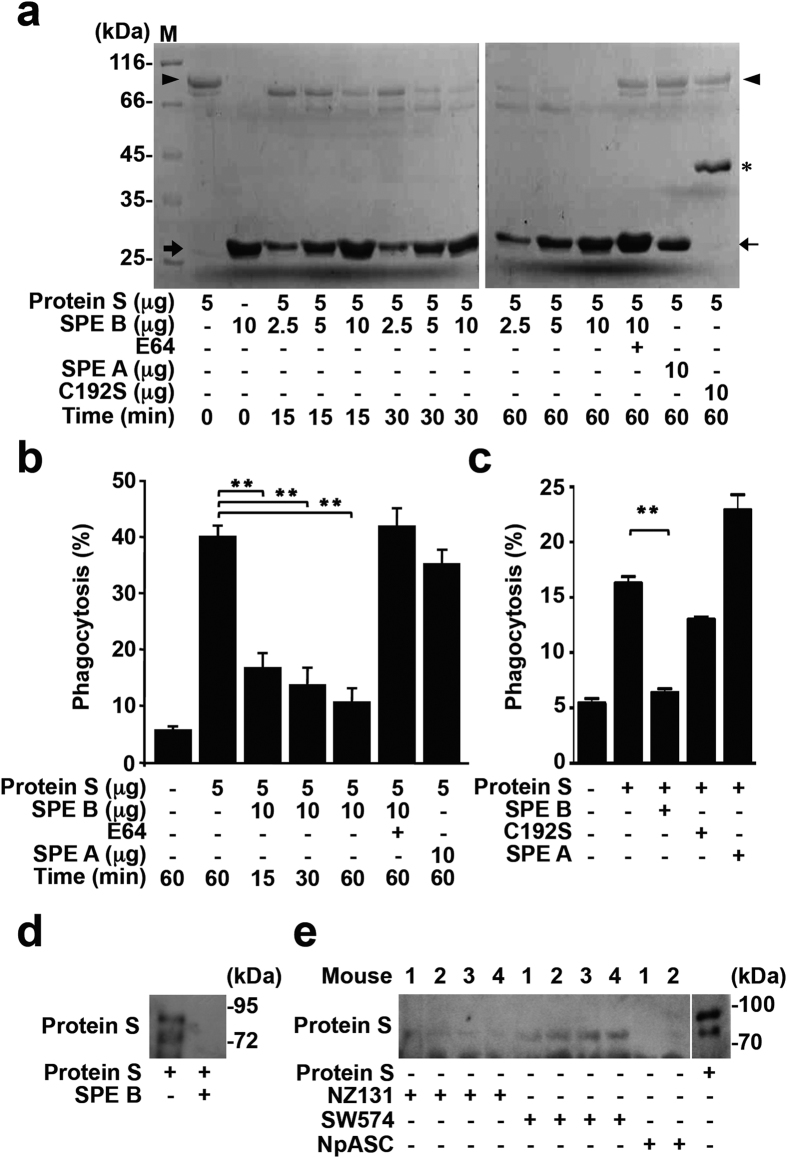
SPE B causes protein S degradation. (**a**) Protein S was incubated with different dosages of SPE B at 37 °C for different time periods as indicated followed by 12.5% SDS-PAGE and Coomassie blue staining. The presence of E64 (16 μM), SPE A, or C192S in protein S cleavage were also detected. The protein expression in different molecular weights is shown. Arrowhead: intact protein S. *The 42 kDa of C192S. Thick arrow: SPE B. Thin arrow: SPE A. (**b**) In the presence or absence of protein S and protein S pretreated with SPE B, SPE A, or SPE B and E64 for different time periods as indicated, macrophages were co-incubated with annxein V/FITC-labeled apoptotic cells for 1 h. The percentages of apoptotic cell engulfment by macrophages were measured by flow cytometric analysis. (**c**) In the presence of protein S (5 μg) and protein S pretreated with 10 μg of SPE B, C192S, or SPE A for 15 min, the percentages of apoptotic cell engulfment by PMA-stimulated THP-1 cells were measured by flow cytometry. Quantified results are shown as mean ± SD of three independent experiments. **P < 0.01 as compared with protein S treatment. (**d**) Purified protein S (5 μg) was incubated with or without SPE B (5 μg) for 30 min, and the expression of protein S was detected using a specific antibody against the C-terminal region of protein S. (**e**) The protein S fragments in the air pouch exudates (20 μl) from mice infected with NZ131 (n = 4), SW574 (n = 4) or NpASC (n = 2) for 48 h were detected by Western blot with a specific antibody against the C-terminal region of protein S. Human plasma-isolated protein S (2 μg) was used as a standard.

According to our findings, the proteolytic activity of SPE B is essential in modulating protein S biofunctions. We therefore determined the protein stability of protein S after co-incubation with SPE B. Results showed that SPE B caused protein S degradation in both time- and dose-dependent manners (Fig. 5a). The molecular weights of protein S precursor and active SPE B were approximately 80 and 28 kDa, respectively. Within 15 min, protein S was cleaved into a ~72 kDa product. Moreover, protein S was fully degraded in the longer incubation time periods. The presence of protease inhibitor E64 significantly blocked this degradation. In contrast to SPE B, SPE A and C192S did not have any effect on protein S degradation. The data thus suggest that SPE B degrades protein S directly and rapidly. We further investigated whether SPE B from the briefer incubation time periods might modulate protein S-stimulated phagocytosis. The results revealed that SPE B but not SPE A markedly reduced protein S-mediated phagocytosis by peritoneal macrophages within 15 min, while E64 could completely block this reaction (Fig. 5b). Similarly, SPE B but not C192S or SPE A reduced protein S-mediated phagocytosis within 15 min in PMA-stimulated THP-1 cells (Fig. 5c). According to these results, SPE B mediates the reduction of efferocytosis through protein S cleavage and the subsequent functional loss of bridging apoptotic cells to macrophages.

SPE B plays a critical role in host protein digestion and in interference of innate immunity. To further confirm the pathogenic effects of SPE B-manipulated protein S cleavage *in vivo*, we examined the protein S fragments in air pouch exudates from mice infected with GAS. By Western blot analysis using specific antibody against C-terminal region of protein S, we first checked that purified protein S was degraded and undetectable by the antibody when co-incubating with SPE B for 30 min (Fig. 5d). Interestingly, similar patterns of protein S degradation could be detected in air pouch exudates from mice infected with wild-type strain NZ131 and *sagB* mutant strain NpASC, whereas *speB* mutant strain SW574 had no effect on protein S expression (Fig. 5e). The full-length protein S can be detected in the air pouch exudates from mice infected with SW574, while barely detectable in the groups infected with NZ131 and NpASC. Our previous study demonstrated that NZ131 and NpASC both possess the complete protease activity of SPE B, but SW574 does not. Mice infected with SW574 showed less tissue damage, lower inflammatory cytokine expression, and a higher survival rate^35^. Accordingly, SPE B-mediated protein S cleavage is speculated to be involved in the pathogenesis of GAS-caused tissue damage, sepsis, and even death.

### The binding of C4BP to apoptotic cells is disrupted by SPE B

**Figure 6:**
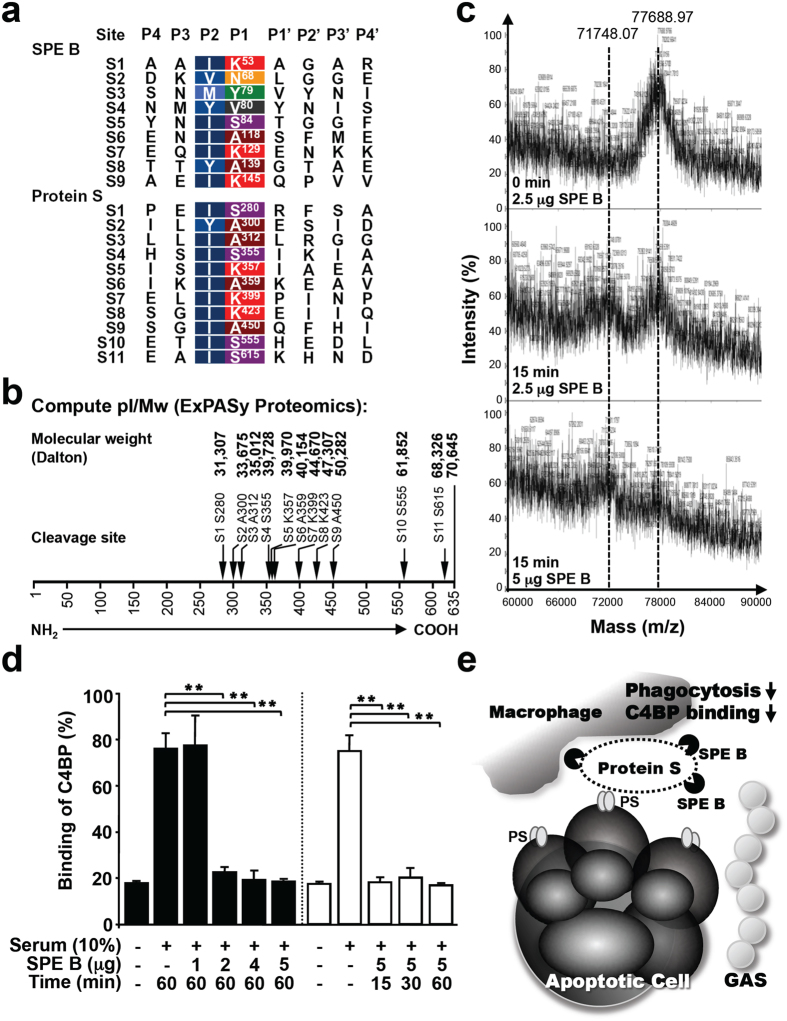
SPE B-mediated protein S cleavage eliminates the binding of C4BP to apoptotic cells. (**a**) Eleven potential recognition (P1 and P2) and cleavage (between P1 and P1’) sites for SPE B on protein S were predicted. (**b**) According to the predicted cleavage site, different molecular weights of degraded protein S are calculated using the program Compute PI/Mw from ExPASy Proteomics as shown. (**c**) Purified human protein S (5 μg) was reacted with different dosages of SPE B for indicated time periods followed by MALDI-TOF MS analysis. The two major peaks of full-length protein S (~77.6 kDa) and cleaved protein S (~71.7 kDa) are shown as dot lines. The range of mass spectrum is shown from m/z 60000 to 90000. (**d**) Human serum was first incubated with different dosages of SPE B for 60 min, or with 5 μg SPE B for different time periods as indicated, which were subsequently co-incubated with STS-stimulated apoptotic cells for 25 min at room temperature. The percentages of C4BP binding to apoptotic cells were measured using specific antibody against C4BP followed by flow cytometric analysis. Data are shown as mean ± SD of three independent experiments. **P < 0.01 as compared with serum treatment. (**e**) GAS-secreted SPE B disrupts the apoptotic cell clearance via modulating protein S cleavage directly. Degraded protein S loses the function as a bridging molecule between macrophages and apoptotic cells. Meanwhile, SPE B-mediated initial cleavage of C-terminal region on protein S and further protein degradation prompt the lost binding of C4BP. The defects in clearance of apoptotic cells and C4BP-mediated complement inhibition may lead to secondary necrosis followed by inflammatory response induction.

To further characterize the effects of SPE B on protein S deregulation, we analyzed the potential cleavage sites of protein S by SPE B as noted in previous studies^36^,^37^. The identification of cleavage sites by SPE B was performed by its autolytic property to generate 28 kDa SPE B (active form) from the 42 kDa precursor. According to the amino acid (aa) sequence that is specifically recognized by active SPE B, 11 potential targeting sites of protein S (shown as S1 to S11) were predicted (Fig. 6a). For instance in S11, SPE B recognizes the site of I614 and S615, and then specifically cleaves at the site between S615 and K616. These cleavage sites located on protein S were identified as similar to the substrates of the papain-like family, with a preference for a hydrophobic residue isoleucine or tyrosine at the P2 site. Based on these cleavage sequences, we used the program of Compute pI/Mw from ExPASy Proteomics^37^ to predict the molecular weights of these cleaved fragments (Fig. 6b). Together with the results obtained from Fig. 5a which indicated that SPE B could cleave protein S into a shorter form (approximate 5–10 kDa less than the precursor) within 15 min, we therefore speculated two potential cleavage sites S10 and S11 on protein S as the initial recognition and cleavage sites of SPE B. Further examination using MALDI-TOF MS analysis indicated that the molecular weight (M.W.) of full-length protein S was ~77.6 kDa while a cleaved form of protein S appeared as ~71.7 kDa after treatment with SPE B for 15 min. A higher dose of SPE B caused the loss of full-length protein S at ~77.6 kDa (Fig. 6c). SPE B-mediated protein S degradation at different time periods was also confirmed by SDS-PAGE (data not shown). Moreover, in addition to human plasma-isolated protein S, C-terminal His tag-conjugated recombinant human protein S was also used for treatment with SPE B followed by His tag detection. SPE B but not C192S or SPE A caused the cleavage of His tag, which suggested that SPE B mediated a rapid C-terminal degradation of protein S (Supplementary Fig. S3). These results suggest that SPE B could rapidly and effectively cleave off a ~5.9 kDa fragment on the C-terminal region of protein S, where S11 might be the first recognition site of SPE B.

In human plasma, protein S forms a complex with C4BP (~60%) through the sex hormone-binding globulin (SHBG)-like domain on the C-terminus^38^,^39^. C4BP binds to the surface of apoptotic cells through protein S of which the N-terminal Gal-residue has highly affinity binding to PS. This binding of C4BP-protein S complex on apoptotic cells provides protection against complement attack^39^,^40^. Deletion of aa 583-635 on protein S may cause a conformational change followed by the loss of binding to C4BP^41^,^42^,^43^. Since SPE B might initially recognize and cleave aa S615 and K616 on the C-terminal region of protein S, the binding of C4BP on apoptotic cells was next determined. By flow cytometric analysis, the expression level of serum-derived C4BP on the surface of apoptotic cells was decreased after serum was pretreated with SPE B (Fig. 6d). These results indicate that the presence of SPE B causes a disruption of apoptotic cell clearance through protein S degradation. The degraded protein S not only loses a bridging function, but also leads to decreased binding of C4BP to apoptotic cells (Fig. 6e). Through these effects, GAS-secreted SPE B may exhibit a new pathologic role in triggering severe inflammation and autoimmune development.

## Discussion

Infection with GAS generally shows a series of inflammatory and autoimmune responses in acute- and post-infection cases^1^,^44^,^45^. However, the immunopathogenesis of GAS infection is not fully defined. In this study, we provide evidence of a novel pathogenic effect of SPE B which involves cleavage of serum-derived vitamin K-dependent protein S followed by inhibition of apoptotic cell clearance by macrophages. The results indicate a decreased apoptotic cell clearance after the C-terminal SHBG-like domain region (aa 616–635) of protein S is initially cleaved by SPE B. Moreover, in association with the cleavage, the adherence of C4BP on apoptotic cell surface was attenuated as well. Previous reports have indicated the importance of the C-terminal region (aa 583–635) of protein S for the maintenance of structural conformation and biofunctions of this protein^41^,^42^,^43^. Therefore, deficient bridging function of protein S is involved in the SPE B-mediated reduction of macrophage efferocytosis. These findings indicate that SPE B may be responsible for the progression of streptococcal diseases associated with inflammation and autoimmunity through the accumulation of non-removed apoptotic cells. Even though there is no direct clinical evidence for the existence of non-removed apoptotic cells in damaged tissues, patients with post-streptococcal glomerulonephritis develop detectable DNA-anti-DNA complex, anti-DNA, and anti-neutrophil cytoplasmic antibodies, which are suggested to be associated with inefficient removal of apoptotic cell fragments^46^,^47^,^48^. Furthermore, our study demonstrates that in NZ131-infected air pouch exudates, the degradation of protein S may correlate with tissue damage and mortality. In addition, previous studies revealed that SPE B can cause high levels of apoptosis in monocytes, epithelial cells, and also in infiltrated neutrophils from mice infected with wild-type GAS strain but not the *speB* mutant strain^15^,^17^,^30^. The induction of apoptosis by GAS-secreted SPE B is not only beneficial for survival of GAS and invasion of the deeper tissues, it is also believed to lead to decreased phagocytosis and further initiate inflammatory responses. SPE B has been shown to degrade a number of proteins including extracellular matrix, cytokines, chemokines, complement, and immunoglobulins^9^. Here we provide the first demonstration that protein S is also a substrate of SPE B and that SPE B-mediated proteolysis of protein S leads to downregulation of efferocytosis.

A number of mechanisms contribute to the promotion of macrophage phagocytosis of apoptotic cells^23^,^24^. The so-called “eat me” signals are activated through exposure of PS on the surface of apoptotic cells to its relative receptor PSR in macrophages. In addition to direct recognition and phagocytosis by macrophage PSR, several bridge proteins including β_2_-glycoprotein I, milk-fat globule epidermal growth factor 8, growth arrest-specific gene 6 (Gas6) and protein S are also involved in mechanisms of apoptotic cell clearance^23^,^26^,^28^,^49^. Based on the genetic characterization, protein S shows ~43% of sequential homology with Gas6 protein, a ligand of Tyro 3/Axl/Mer (TAM) expressed on the surface of macrophages^25^,^26^,^27^,^50^,^51^. The recognition and the binding of Gas6 to PS and TAM effectively trigger PI3K/Akt activation followed by promoting cell proliferation, cytoskeletal reorganization, engulfment, and subsequent anti-inflammatory macrophage polarization^52^,^53^. Protein S, similar to Gas6, plays a central role in serum-stimulated enhancement of macrophage phagocytosis of apoptotic cells. It has a high affinity with PS via the N-terminal Gal-residues, and binds to the relative receptors Tyro3/Mer expressed on the surface of macrophages by the two C terminal laminin G regions that are homologous to the SHBG domains^25^,^27^,^51^,^54^. In this study, we demonstrated that SPE B cleaves protein S leading to a decrease of apoptotic cell clearance. Interestingly, the downregulation of protein S-mediated phagocytosis occurred within 15 min after SPE B treatment. By proteomic prediction and MALDI-TOF Mass analysis, the C-terminal fragment (aa 614–615) of protein S likely contains the initial recognition and cleavage site for SPE B. Moreover, results obtained from secondary structural prediction indicated that S8 K423 and S11 S615 are located at two loop domains on the SHBG-like domain of protein S (data not shown). According to previous reports, deletion of the C-terminal SHBG-like domain leads to a major conformational change on protein S^41^,^42^. Therefore, we speculated that the initial cleavage event mediated by SPE B might cause a disruption of protein S bridging apoptotic cells to macrophages followed by inhibition of engulfment. Recent evidence indicated that Mer acts as a tolerogenic receptor in resting macrophages, whereas Axl primarily works in the feedback inhibition of inflammation^53^. Since SPE B efficiently cleaves protein S, it is intriguing to determine whether SPE B also causes an effect on Gas6. The distinct regulations of SPE B on protein S and Gas6, as well as tolerogenic and activated macrophage-mediated efferocytosis need further investigation.

Opsonization is the innate immunity process where microorganisms and apoptotic cells become coated with molecules that allow them to bind to receptors on phagocytes. Apoptotic cells are opsonized with protein S to promote engulfment by phagocytes. Meanwhile, to avoid the induction of inflammation that results from secondary necrosis, early apoptotic cells are immediately opsonized with C4BP, a well-known inhibitor of complement. In the circulation, protein S is abundantly expressed and complexed with C4BP. C4BP binds to apoptotic cells and offers protection from complement attack through association with protein S^39^,^40^. Our present results demonstrate the cleavage of protein S by SPE B decreases the binding ability of C4BP to apoptotic cells, and thus destroys the protective effect of C4BP. The three domains (aa 420–434, 447–460 and 583–635) that are located on the C-terminal SHBG-like domain of protein S are known as C4BP-binding regions^38^,^40^,^41^,^42^,^43^,^55^. In particular, aa 605–614 in protein S is proposed to be most important for its interaction with C4BP. SPE B-initiated protein S cleavage on S8 K423, S9 A450 and S11 S615 are located on C4BP-binding regions. GAS-secreted SPE B not only disrupts protein S-mediated efferocytosis, it also avoids protection provided by C4BP. Moreover, other studies showed that streptococcal M protein binds to C4BP to escape innate immunity by phagocytosis resistance and preventing complement attack as well^1^,^39^,^56^. SPE B-elicited dissociation of protein S and C4BP is therefore speculated to be beneficial for free-C4BP binding to M proteins on GAS surface and results in a decrease of opsonophagocytosis.

Phagocytosis of apoptotic cells is a routine function by macrophages in innate immunity. To date, there are still no well-characterized mechanisms to explore the relationship of macrophage phagocytisis to immunopathogenesis of GAS infection. Previous studies showed that SPE B can cause direct cell damage and indirect effects by causing immunoglobulin, fibronectin, vitronectin and properdin cleavage. The involvement of fibronectin in the apoptotic cell clearance has been demonstrated as well^57^. In addition to protein S, it is possible that SPE B initiates a reduction of efferocytosis through manipulating fibronectin degradation. This question needs further investigation. To the best of our knowledge, this is the first demonstration of disruption of apoptotic cell clearance by SPE B-stimulated protein S cleavage. For future therapeutic implications, SPE B inactivation may be a potential strategy to rescue the deregulation of innate immunity in GAS infection by sustaining the clearance of apoptotic cells by macrophages.

## Materials and Methods

### Mice

BALB/c and BALB/cByJNarl breeder mice were obtained from The Jackson Laboratory (Bar Harbor, Maine, USA) and the National Laboratory Animal Center in Taiwan, respectively. They were maintained on standard laboratory food and water ad libitum in the animal centers at the National Cheng Kung University and I-Shou University. Their 8-wk-old progeny were used for the purification of residential peritoneal macrophages and air pouch infectious model in this study. Animal handling and procedures were reviewed and approved by the Institutional Animal Care and Use Committee (IACUC) of National Cheng Kung University, and conducted in accordance with the Guidelines for Committee of Laboratory Care and Use, National Cheng Kung University.

### Cell cultures, antibodies, and reagents

Peritoneal macrophages were purified from BALB/c mice according to the previous method with modification^58^. We injected 5 ml of PBS into the mouse peritoneum and then aspirated fluid from peritoneum after 5 min post-injection. After washing with PBS, isolated cells were seeded and grown in RPMI 1640 culture medium (Gibco BRL, Grand Island, NY, USA) containing 10% heat-inactivated FCS, 2 mM L-glutamate, and 50 μg/ml of gentamicin. By flow cytometric analysis, more than 95% were CD11b-positive cells used in this study. The human monocytic THP-1 cells obtained from the American Type Culture Collection (Manassas, VA, USA) were grown in RPMI 1640 medium containing 10% FCS, L-glutamate and gentamicin, and further cultured with 160 nM phorbol myristate acetate (PMA) for 48 h to induce a macrophage-like phenotype. The human lymphoid T cell line Jurkat was obtained from Dr. B. C. Yang (Department of Microbiology and Immunology, National Cheng Kung University, Taiwan). Jurkat T cells were cultured in RPMI 1640 culture medium and used for the preparation of apoptotic cells. Purified human protein S was purchased from Calbiochem (San Diego, CA, USA). The rabbit antibody specific for human protein S was purchased from DAKO (Glostrup, Denmark). The polyclonal goat anti-human C4BP, anti-human protein S (C-terminal), and rabbit anti-mouse CD11b were from Santa Cruz Biotechnology (Santa Cruz, CA, USA). FITC-conjugated anti-human CD11b and anti-human CD36 were purchased from eBioscience (San Diego, CA, USA). FITC-conjugated goat anti-rabbit and donkey anti-goat IgG were obtained from The Jackson Laboratory (Bar Harbor, ME, USA). The rabbit antibody against His tag and HRP-conjugated goat anti-rabbit antibody were purchased from Abcam (Cambridge, MA, USA). The protease inhibitor E64, PMA, and staurosporine (STS) were obtained from Sigma-Aldrich (St. Louis, MO, USA). The normal human serum was collected by the ethical approval from the Institutional Review Board of National Cheng Kung University Hospital, No. ER-98-287, with the written informed consent obtained from healthy volunteers.

### Bacterial culture supernatant

GAS NZ131 (type M49, T14 strain) was a gift from Dr. D. R. Martin, New Zealand Communicable Disease Center, Porirua. Disruption of *speB* gene to generate *speB* mutant strain, SW510 and SW574, and disruption of *sagB* gene to generate *sagB* isogenic mutant strain, NpASC, were previously described^35^,^59^. For the collection of SPE B-containing bacterial culture supernatants, the fresh colony of NZ131 and SW510 were grown in RPMI 1640 culture medium at 37 °C for 18 h. After centrifugation, the supernatants were harvested and stored at −70 °C.

### Purification of recombinant proteins

For the expression of recombinant SPE A, SPE B, and protease-negative mutated protein C192S, the construction and purification of these proteins were performed as previously described^18^. Briefly, the genes of SPE A, SPE B, and C192S were PCR-amplified and cloned into the pET21a vector, which were then transformed into the *Escherichia coli* BL21(DE3) pLys strain. After incubation in Luria-Bertani medium containing ampicillin (100 μg/ml) and induction by isopropyl-D-thiogalactopyranoside (IPTG; 100 mg/ml), cells were harvested and lysed using a French press at 1,500 kg/cm^2^ for 30 s. The collected supernatants were then purified by the Ni-column (Amersham Pharmacia Biotech, Piscataway, NJ, USA) separation and imidazole gradient (0–200 mM) elution. The collected fractions were concentrated by Amicon ultrafiltration with a 10-kDa cutoff membrane and equilibrated with PBS. Purified SPE A, SPE B and C192S were passed through Detoxi-Gel (Pierce, Rockford, IL, USA) to remove possible lipopolysaccharide contamination. The preparations were then subjected to testing for endotoxin contamination using a Limulus amebocyte lysate assay (Pyrotell, Associates of Cape Cod, Falmouth, MA, USA), and the endotoxin concentrations of all purified proteins were <0.5 EU/ml. The purity was evaluated by SDS-PAGE, and the protease activity was detected by adding 100 μl of purified SPE B or C192S to 200 μl of azocasein solution (50 mM Tris-HCl [pH 8.0], 10 mM dithiothreitol, 5 mM EDTA) for 30 min at 37 °C. The reaction was stopped by adding 100 μl of 15% TCA for 15 min on ice. The mixture was then centrifuged, and an equal volume of 0.5 N NaOH was added to the supernatant. The absorbance of the sample was measured with a microplate reader at the wavelength of 450 nm (Emax microplate reader; Molecular Devices, Palo Alto, CA, USA). Purified proteins were stored at −70 °C in aliquots for use in the experiments.

### Apoptotic cells and phagocytosis

Human Jurkat T cells (10^6 ^cells/ml) were treated with 100 μM of STS in RPMI 1640 culture medium for 12 h at 37 °C. STS-treated apoptotic cells were labeled using FITC-conjugated annexin V staining (Biovision, Mountain View, CA, USA). Around 80% of the cells were annexin V-positive cells after STS treatment. To measure the phagocytosis of apoptotic cells, 10% FCS, 10% human serum, and protein S were first incubated with or without recombinant SPE B, C192S, and SPE A for indicated time periods at 37 °C followed by addition of protease inhibitor E64 (16 μM) to stop the proteolytic reaction. These mixtures were then co-cultured with purified murine peritoneal macrophages or PMA-stimulated THP-1 cells (10^6^ cells) and annexin V-labeled apoptotic Jurkat T cells (2 × 10^6^ cells) for 1 h at 37 °C. For confocal laser microscopic (Leica TCS SPII, Nussloch, Germany) and wide-field DeltaVision deconvolution microscopic (Applied Precision, GE Healthcare Life Science, Issaquah, WA, USA) observation, peritoneal macrophages and PMA-stimulated THP-1 cells were further stained with MitoTracker-Red (Molecular Probes, Leiden, The Netherlands) and DAPI (Calbiochem). The stacks of fluorescence images from deconvolution microscope were acquired and deconvoluted using SoftWorx software, and later analyzed with Volocity software (Perkin-Elmer, Waltham, MA, USA). For flow cytometric analysis, the cells were washed, detached, and analyzed by flow cytometry (FACSCalibur; BD Biosciences, San Jose, CA, USA) with excitation set at 488 nm. The emission was detected with the FL-1 channel followed by CellQuest Pro 4.0.2 software (BD Biosciences) analysis, and quantification was done using WinMDI 2.8 software (The Scripps Institute).

### The air pouch exudates collection

Mice were injected subcutaneously with 2 ml of air to form an air pouch, and 0.2 ml of the bacterial suspension containing 3 × 10^8^ colony forming units (CFU) of NZ131 or its isogenic mutants was then inoculated into the air pouch. The air pouch exudates were collected at 48 h post-infection and stored at −70 °C until use.

### Protein S and C4BP binding assay

Human serum or protein S were pretreated with SPE B, C192S or bacterial culture supernatants for indicated time periods at 37 °C followed by E64 (16 μM) addition to stop the proteolytic reaction. Samples were then incubated with STS (100 μM)-stimulated apoptotic Jurkat T cells (10^6^) for 25 min at room temperature. After washing twice, cells were fixed with 1% formaldehyde in PBS for 10 min, and then washed again with PBS. Fixed cells were incubated with specific antibodies against human protein S (DAKO) and human C4BP (Santa Cruz Biotechnology), respectively, at 4 °C for 30 min followed by FITC-conjugated goat anti-rabbit or donkey anti-goat IgG staining, and analyzed by flow cytometry (FACSCalibur; BD Biosciences) with excitation set at 488 nm. The emission was detected with the FL-1 channel followed by CellQuest Pro 4.0.2 software (BD Biosciences) analysis, and quantification was done using WinMDI 2.8 software (The Scripps Institute).

### Protein electrophoresis and Western blot analysis

The reactive proteins were diluted in Tris-HCl loading buffer (final buffer concentration approximately 62.5 mM Tris-HCl [pH 6.8], 2% SDS, 10% glycerol, 5% 2-mercaptoethanol, 0.001% bromophenol) and boiled for 10 min at 95 °C. Samples were subjected to electrophoresis on 10% polyacrylamide gels at 100 V for 100 min in Tris-glycine running buffer (25 mM Tris, 192 mM glycine, 0.1% SDS, pH 8.3). Proteins were determined by gel staining with a Coomassie brilliant blue solution (0.25% Coomassie blue R-250, 10% acetic acid, 45% ethanol) followed by destaining with a buffer containing 10% acetic acid and 10% methanol. For Western blot analysis, proteins were resolved by SDS-PAGE and then transferred to a PVDF membrane (Millipore Corporation, Billerica, MA, USA). After blocking, blots were incubated with a specific antibody against C-terminal region of protein S (Santa Cruz Biotechnology) followed by HRP-conjugated donkey anti-goat IgG (Santa Cruz Biotechnology) incubation, and subsequently developed using an ECL Western blot detection kit (Millipore Corporation) according to the manufacturer’s instructions.

### Cleavage site and molecular weight prediction

Analysis of cleavage sites of protein S by SPE B was performed as referred to previous studies^36^,^37^. The sequence of protein S was obtained from NCBI Protein Database accession number NP_000304 (http://www.ncbi.nlm.nih.gov) and initially cloned and characterized by Hoskins *et al*.^60^. Based on results obtained from cleavage site analysis, the molecular weights of SPE B-cleaved proteins were computed using the Expert Protein Analysis System proteomics server of the Swiss Institute of Bioinformatics (http://expasy.hcuge.ch).

### Mass analysis

Human plasma-isolated protein S (5 μg) was incubated with different doses of SPE B for the indicated time periods followed by addition of E64 to stop the reaction. The protein samples (0.5 μl) were mixed with matrix solution (0.5 μl, 10 mg/ml sinapinic acid in 50% acetonitrile and 0.1% trifluoroacetic acid in deionized H_2_O) and then were spotted onto stainless steel plates followed by air-dried before subsequent MALDI-TOF MS analysis (4800 ABSciex, Framingham, MA, USA). Data were processed and analyzed by Data Explorer software.

### Statistics

Comparisons between various treatments were performed by unpaired *t*-test with GraphPad Prism version 6.0 (La Jolla, CA, USA). Statistical significance was set at *P* < 0.05.

## Additional Information

**How to cite this article**: Chen, C.-L. *et al*. Streptococcal pyrogenic exotoxin B inhibits apoptotic cell clearance by macrophages through protein S cleavage. *Sci. Rep*. **6**, 26026; doi: 10.1038/srep26026 (2016).

## Supplementary Material

Supplementary Information

## References

[b1] Walker M. J. . Disease manifestations and pathogenic mechanisms of group A streptococcus. Clin. Microbiol. Rev. 27, 264–301 (2014). 24696436 10.1128/CMR.00101-13 PMC3993104

[b2] Cole J. N., Barnett T. C., Nizet V. & Walker M. J. Molecular insight into invasive group A streptococcal disease. Nat. Rev. Microbiol. 9, 724–736 (2011). 21921933 10.1038/nrmicro2648

[b3] Hsueh P. R. . Invasive group A streptococcal disease in Taiwan is not associated with the presence of streptococcal pyrogenic exotoxin genes. Clin. Infect. Dis. 26, 584–589 (1998). 9524827 10.1086/514567

[b4] Kuo C. F. . Role of streptococcal pyrogenic exotoxin B in the mouse model of group A streptococcal infection. Infect. Immun. 66, 3931–3935 (1998). 9673282 10.1128/iai.66.8.3931-3935.1998 PMC108455

[b5] Kuo C. F. . Histopathologic changes in kidney and liver correlate with streptococcal pyrogenic exotoxin B production in the mouse model of group A streptococcal infection. Microb. Pathog. 36, 273–385 (2004). 15043862 10.1016/j.micpath.2004.01.003

[b6] Kansal R. G., McGeer A., Low D. E., Norrby-Teglund A. & Kotb M. Inverse relation between disease severity and expression of the streptococcal cysteine protease, SpeB, among clonal M1T1 isolates recovered from invasive group A streptococcal infection cases. Infect. Immun. 68, 6362–6369 (2000). 11035746 10.1128/iai.68.11.6362-6369.2000 PMC97720

[b7] Krishnan K. C., Mukundan S., Figueroa J. A. L., Caruso J. A. & Kotb M. Metal-mediated modulation of streptococcal cysteine protease activity and its biological implications. Infect. Immun. 82, 2992–3001 (2014). 24799625 10.1128/IAI.01770-14 PMC4097633

[b8] Friães A., Pato C., Melo-Cristino J. & Ramirez M. Consequences of the variability of the CovRS and RopB regulators among *Streptococcus pyogenes* causing human infections. Sci. Rep. 5, 12057 (2015). 26174161 10.1038/srep12057 PMC4502508

[b9] Nelson D. C., Garbe J. & Collin M. Cysteine proteinase SpeB from *Streptococcus pyogenes* - a potent modifier of immunologically important host and bacterial proteins. Biol. Chem. 392, 1077–1088 (2011). 22050223 10.1515/BC.2011.208

[b10] Sumitomo T., Nakata M., Higashino M., Terao Y. & Kawabata S. Group A streptococcal cysteine protease cleaves epithelial junctions and contributes to bacterial translocation. J. Biol. Chem. 288, 13317–13324 (2013). 23532847 10.1074/jbc.M113.459875 PMC3650370

[b11] Collin M. . EndoS and SpeB from *Streptococcus pyogenes* inhibit immunoglobulin-mediated opsonophagocytosis. Infect. Immun. 70, 6646–6651 (2002). 12438337 10.1128/IAI.70.12.6646-6651.2002 PMC133027

[b12] Von Pawel-Rammingen U. & Björck L. IdeS and SpeB: immunoglobulin-degrading cysteine proteinases of *Streptococcus pyogenes*. Curr. Opin. Microbiol. 6, 50–55 (2003). 12615219 10.1016/s1369-5274(03)00003-1

[b13] Tsao N. . Streptococcal pyrogenic exotoxin B cleaves properdin and inhibits complement-mediated opsonophagocytosis. Biochem. Biophys. Res. Commun. 339, 779–784 (2006). 16329996 10.1016/j.bbrc.2005.11.078

[b14] Kuo C. F., Lin Y. S., Chuang W. J., Wu J. J. & Tsao N. Degradation of complement 3 by streptococcal pyrogenic exotoxin B inhibits complement activation and neutrophil opsonophagocytosis. Infect. Immun. 76, 1163–1169 (2008). 18174338 10.1128/IAI.01116-07 PMC2258837

[b15] Lukomski S. . Genetic inactivation of an extracellular cysteine protease (SpeB) expressed by *Streptococcus pyogenes* decreases resistance to phagocytosis and dissemination to organs. Infect. Immun. 66, 771–776 (1998). 9453640 10.1128/iai.66.2.771-776.1998 PMC107969

[b16] Lukomski S. . Extracellular cysteine protease produced by *Streptococcus pyogenes* participates in the pathogenesis of invasive skin infection and dissemination in mice. Infect. Immun. 67, 1779–1788 (1999). 10085018 10.1128/iai.67.4.1779-1788.1999 PMC96528

[b17] Kuo C. F. . Streptococcal pyrogenic exotoxin B induces apoptosis and reduces phagocytic activity in U937 cells. Infect. Immun. 67, 126–130 (1999). 9864206 10.1128/iai.67.1.126-130.1999 PMC96287

[b18] Tsai W. H. . Streptococcal pyrogenic exotoxin B-induced apoptosis in A549 cells is mediated by a receptor- and mitochondrion-dependent pathway. Infect. Immun. 72, 7055–7062 (2004). 15557629 10.1128/IAI.72.12.7055-7062.2004 PMC529174

[b19] Savill J., Dransfield I., Gregory C. & Haslett C. A blast from the past: clearance of apoptotic cells regulates immune responses. Nat. Rev. Immunol. 2, 965–975 (2002). 12461569 10.1038/nri957

[b20] Kimani S. G. . Contribution of defective PS recognition and efferocytosis to chronic inflammation and autoimmunity. Front. Immunol. 5, 566 (2014). 25426118 10.3389/fimmu.2014.00566 PMC4226236

[b21] Szondy Z., Garabuczi E., Joós G., Tsay G. J. & Sarang Z. Impaired clearance of apoptotic cells in chronic inflammatory diseases: therapeutic implications. Front. Immunol. 5, 354 (2014). 25136342 10.3389/fimmu.2014.00354 PMC4117929

[b22] Hart S. P., Smith J. R. & Dransfield I. Phagocytosis of opsonized apoptotic cells: roles for ‘old-fashioned’ receptors for antibody and complement. Clin. Exp. Immunol. 135, 181–185 (2004). 14738443 10.1111/j.1365-2249.2003.02330.x PMC1808943

[b23] Wu Y., Tibrewal N. & Birge R. B. Phosphatidylserine recognition by phagocytes: a view to a kill. Trends Cell Biol. 16, 189–197 (2006). 16529932 10.1016/j.tcb.2006.02.003

[b24] Poon I. K., Lucas C. D., Rossi A. G. & Ravichandran K. S. Apoptotic cell clearance: basic biology and therapeutic potential. Nat. Rev. Immunol. 14, 166–180 (2014). 24481336 10.1038/nri3607 PMC4040260

[b25] Anderson H. A. . Serum-derived protein S binds to phosphatidylserine and stimulates the phagocytosis of apoptotic cells. Nat. Immunol. 4, 87–91 (2003). 12447359 10.1038/ni871

[b26] Scott R. S. . Phagocytosis and clearance of apoptotic cells is mediated by MER. Nature 411, 207–211 (2001). 11346799 10.1038/35075603

[b27] Anderson H. A. & Shacter E. Natural anticoagulant proteins in the regulation of autoimmunity: potential role of protein S. Curr. Pharm. Des. 10, 929–937 (2004). 15032696 10.2174/1381612043452839

[b28] Roos A. . Mini-review: A pivotal role for innate immunity in the clearance of apoptotic cells. Eur. J. Immunol. 34, 921–929 (2004). 15048702 10.1002/eji.200424904

[b29] Goldmann O., Rohde M., Chhatwal G. S. & Medina E. Role of macrophages in host resistance to group A streptococci. Infect. Immun. 72, 2956–2963 (2004). 15102808 10.1128/IAI.72.5.2956-2963.2004 PMC387899

[b30] Tsai P. J., Lin Y. S., Kuo C. F., Lei H. Y. & Wu J. J. Group A streptococcus induces apoptosis in human epithelial cells. Infect. Immun. 67, 4334–4339 (1999). 10456871 10.1128/iai.67.9.4334-4339.1999 PMC96749

[b31] Cywes-Bentley C., Hakansson A., Christianson J. & Wessels M. R. Extracellular group A *Streptococcus* induces keratinocyte apoptosis by dysregulating calcium signalling. Cell. Microbiol. 7, 945–955 (2005). 15953027 10.1111/j.1462-5822.2005.00525.x

[b32] Aikawa C. . Reactive oxygen species induced by *Streptococcus pyogenes* invasion trigger apoptotic cell death in infected epithelial cells. Cell. Microbiol. 12, 814–830 (2010). 20070306 10.1111/j.1462-5822.2010.01435.x

[b33] Stepczynska A. . Staurosporine and conventional anticancer drugs induce overlapping, yet distinct pathways of apoptosis and caspase activation. Oncogene 20, 1193–1202 (2001). 11313863 10.1038/sj.onc.1204221

[b34] Mevorach D., Mascarenhas J. O., Gershov D. & Elkon K. B. Complement-dependent clearance of apoptotic cells by human macrophages. J. Exp. Med. 188, 2313–2320 (1998). 9858517 10.1084/jem.188.12.2313 PMC2212421

[b35] Hung C. H. . Synergistic effects of streptolysin S and streptococcal pyrogenic exotoxin B on the mouse model of group A streptococcal infection. Med. Microbiol. Immunol. 201, 357–369 (2012). 22610375 10.1007/s00430-012-0241-6

[b36] Doran J. D. . Autocatalytic processing of the streptococcal cysteine protease zymogen: processing mechanism and characterization of the autoproteolytic cleavage sites. Eur. J. Biochem. 263, 145–151 (1999). 10429198 10.1046/j.1432-1327.1999.00473.x

[b37] Chen C. Y. . Maturation processing and characterization of streptopain. J. Biol. Chem. 278, 17336–17343 (2003). 12621045 10.1074/jbc.M209038200

[b38] Webb J. H., Blom A. M. & Dahlbäck B. Vitamin K-dependent protein S localizing complement regulator C4b-binding protein to the surface of apoptotic cells. J. Immunol. 169, 2580–2586 (2002). 12193728 10.4049/jimmunol.169.5.2580

[b39] Blom A. M., Villoutreix B. O. & Dahlbäck B. Complement inhibitor C4b-binding protein-friend or foe in the innate immune system? Mol. Immunol. 40, 1333–1346 (2004). 15072852 10.1016/j.molimm.2003.12.002

[b40] Webb J. H., Blom A. M. & Dahlbäck B. The binding of protein S and the protein S-C4BP complex to neutrophils is apoptosis dependent. Blood Coagul. Fibrinolysis 14, 355–359 (2003). 12945877 10.1097/00001721-200306000-00006

[b41] Chang G. T. . Studies of the interaction between human protein S and human C4b-binding protein using deletion variants of recombinant human protein S. Thromb. Haemost. 71, 461–467 (1994). 8052964

[b42] Villoutreix B. O., Dahlbäck B., Borgel D., Gandrille S. & Muller Y. A. Three-dimensional model of the SHBG-like region of anticoagulant protein S: new structure-function insights. Proteins 43, 203–216 (2001). 11276089

[b43] Rezende S. M., Simmonds R. E. & Lane D. A. Coagulation, inflammation, and apoptosis: different roles for protein S and the protein S-C4b binding protein complex. Blood 103, 1192–1201 (2004). 12907438 10.1182/blood-2003-05-1551

[b44] Fieber C. & Kovarik P. Responses of innate immune cells to group A Streptococcus. Front. Cell. Infect. Microbiol. 4, 140 (2014). 25325020 10.3389/fcimb.2014.00140 PMC4183118

[b45] Chakravarty S. D., Zabriskie J. B. & Gibofsky A. Acute rheumatic fever and streptococci: the quintessential pathogenic trigger of autoimmunity. Clin. Rheumatol. 33, 893–901 (2014). 24894108 10.1007/s10067-014-2698-8

[b46] Vilches A. R. & Williams D. G. Persistent anti-DNA antibodies and DNA-anti-DNA complexes in post-streptococcal glomerulonephritis. Clin. Nephrol. 22, 97–101 (1984). 6332702

[b47] Ardiles L. G., Valderrama G., Moya P. & Mezzano S. A. Incidence and studies on antigenic specificities of antineutrophil-cytoplasmic autoantibodies (ANCA) in poststreptococcal glomerulonephritis. Clin. Nephrol. 47, 1–5 (1997). 9021233

[b48] Rodríguez-Iturbe B. & Batsford S. Pathogenesis of poststreptococcal glomerulonephritis a century after Clemens von Pirquet. Kidney Int. 71, 1094–1104 (2007). 17342179 10.1038/sj.ki.5002169

[b49] Hanayama R. . Identification of a factor that links apoptotic cells to phagocytes. Nature 417, 182–187 (2002). 12000961 10.1038/417182a

[b50] Ishimoto Y., Ohashi K., Mizuno K. & Nakano T. Promotion of the uptake of PS liposomes and apoptotic cells by a product of growth arrest-specific gene, gas6. J. Biochem. 127, 411–417 (2000). 10731712 10.1093/oxfordjournals.jbchem.a022622

[b51] Lemke G. & Burstyn-Cohen T. TAM receptors and the clearance of apoptotic cells. Ann. N Y Acad. Sci. 1209, 23–29 (2010). 20958312 10.1111/j.1749-6632.2010.05744.x PMC3061224

[b52] Tibrewal N. . Autophosphorylation docking site Tyr-867 in Mer receptor tyrosine kinase allows for dissociation of multiple signaling pathways for phagocytosis of apoptotic cells and down-modulation of lipopolysaccharide-inducible NF-κB transcriptional activation. J. Biol. Chem. 283, 3618–3627 (2008). 18039660 10.1074/jbc.M706906200

[b53] Alciato F., Sainaghi P. P., Sola D., Castello L. & Avanzi G. C. TNF-α, IL-6, and IL-1 expression is inhibited by GAS6 in monocytes/macrophages. J. Leukoc. Biol. 87, 869–875 (2010). 20103767 10.1189/jlb.0909610

[b54] Zagórska A., Través P. G., Lew E. D., Dransfield I. & Lemke G. Diversification of TAM receptor tyrosine kinase function. Nat. Immunol. 15, 920–928 (2014). 25194421 10.1038/ni.2986 PMC4169336

[b55] Walker F. J. Characterization of a synthetic peptide that inhibits the interaction between protein S and C4b-binding protein. J. Biol. Chem. 264, 17645–17648 (1989). 2530213

[b56] Thern A., Stenberg L., Dahlbäck B. & Lindahl G. Ig-binding surface proteins of *Streptococcus pyogenes* also bind human C4b-binding protein (C4BP), a regulatory component of the complement system. J. Immunol. 154, 375–386 (1995). 7995956

[b57] McCutcheon J. C. . Regulation of macrophage phagocytosis of apoptotic neutrophils by adhesion to fibronectin. J. Leukoc. Biol. 64, 600–607 (1998). 9823764 10.1002/jlb.64.5.600

[b58] Lovett D. H. . Interleukin 1 and the glomerular mesangium. I. Purification and characterization of a mesangial cell-derived autogrowth factor. J. Immunol. 136, 3700–3705 (1986). 3486219

[b59] Tsai P. J. . Effect of group A streptococcal cysteine protease on invasion of epithelial cells. Infect. Immun. 66, 1460–1466 (1998). 9529068 10.1128/iai.66.4.1460-1466.1998 PMC108075

[b60] Hoskins J., Norman D. K., Beckmann R. J. & Long G. L. Cloning and characterization of human liver cDNA encoding a protein S precursor. Proc. Natl. Acad. Sci. USA 84, 349–353 (1987). 3467362 10.1073/pnas.84.2.349 PMC304204

