# Supplementary material for: Streptococcal pyrogenic exotoxin B inhibits apoptotic cell clearance by macrophages through protein S cleavage
Source: Sci Rep. 2016 May 16;6:26026. doi: 10.1038/srep26026 (PMC4867609; doi:10.1038/srep26026)
Supplement: Supplementary Information [file srep26026-s1.doc]

Supplementary information for

**Streptococcal Pyrogenic Exotoxin B Inhibits Apoptotic Cell Clearance by Macrophages through Protein S Cleavage**

Chia-Ling Chen^1*^, Yueh-Ying Wu^2*^, Chiou-Feng Lin^3,4^, Chih-Feng Kuo^5^, Chia-Li Han^6^, Shuying Wang^2,7^, Woei-Jer Chuang^7,8^, Chiu-Yueh Chen^8^, Jiunn-Jong Wu^7,9^, Pei-Jane Tsai^7,9^, Ching-Chuan Liu^7,10^ & Yee-Shin Lin^2,7**^

**Supplementary Figure 1. Detection of apoptotic cells, peritoneal macrophages, and PMA-stimulated THP-1 cells.** (a) Human Jurkat T cells were treated with or without 100 μM STS for 12 h and subsequently labeled with FITC-conjugated annexin V followed by flow cytometric analysis. (b) Peritoneal macrophages were isolated from BALB/c mice and incubated at 37°C for 3 h. The purity of peritoneal macrophages were then stained with or without a specific antibody against CD11b followed by flow cytometric analysis. (c) THP-1 cells were stimulated with PMA (160 nM) for 48 h followed by the measurement of differential markers, FITC-conjugated CD11b and CD36.

**Supplementary Figure 2. SPE A causes no effect on protein S-mediated efferocytosis.** PMA-stimulated THP-1 cells (*Red*) were incubated with FITC-labeled apoptotic cells (*Green*) in the presence of protein S or SPE A-pretreated protein S followed by confocal microscopic observation. Nuclei were stained with DAPI (*Blue*). The scale bar is 10 μm.

**Supplementary Figure 3. SPE B digests protein S-His.** Recombinant protein S-His was incubated with purified SPE B, C192S, or SPE A for 15 min followed by Western blot analysis.
